# Comparison of antithrombin activity assays in detection of patients with heparin binding site antithrombin deficiency: systematic review and meta-analysis

**DOI:** 10.1038/s41598-023-43941-x

**Published:** 2023-10-04

**Authors:** Tamara Rojnik, Nataša Sedlar, Nana Turk, Andrej Kastrin, Maruša Debeljak, Mojca Božič Mijovski

**Affiliations:** 1 Laboratory for Haemostasis and Atherothrombosis, Department of Vascular Diseases, University Medical Centre Ljubljana, 1000 Ljubljana, Slovenia; 2 Faculty of Pharmacy, University of Ljubljana, 1000 Ljubljana, Slovenia; 3 Central Medical Library, Faculty of Medicine, University of Ljubljana, 1000 Ljubljana, Slovenia; 4 Faculty of Medicine, Institute for Biostatistics and Medical Informatics, University of Ljubljana, 1000 Ljubljana, Slovenia; 5 Clinical Institute for Special Laboratory Diagnostics, University Children’s Hospital, University Medical Centre Ljubljana, 1000 Ljubljana, Slovenia; 6 Department of Paediatrics, Faculty of Medicine, University of Ljubljana, 1000 Ljubljana, Slovenia

**Keywords:** Blood proteins, Screening, Rare variants, Thromboembolism, Molecular medicine

## Abstract

Antithrombin (AT) deficiency increases the risk for venous thromboembolism, therefore, a highly sensitive assay to identify this condition is crucial. The aim of this paper was to perform a meta-analysis comparing AT activities measured by different AT activity assays in patients with heparin binding site AT deficiency. In addition, the diagnostic sensitivity of selected assays was compared depending on the available data. An extensive literature search was performed considering results with publication date up to July 10, 2021. Seven relevant English-language observational studies, comparing AT activity measured by different AT activity assays in Caucasian Europeans with either the AT Budapest III or AT Padua I mutation were included in meta-analyses. There was no significant difference in AT activity between Labexpert and Innovance in patients with AT Budapest III (P = 0.567) and AT Padua I (P = 0.265), while AT activity determined by HemosIL was significantly higher compared to Innovance for both mutations (AT Budapest III: P < 0.001; AT Padua I: P < 0.001). These results are in line with the results of comparison of diagnostic sensitivity. In patients with AT Budapest III, the AT activity was also higher when measured with Berichrom compared to Innovance (P = 0.002), however, the results of comparison of diagnostic sensitivity across studies were variable. No significant difference (P = 0.117) in AT activity as well as diagnostic sensitivity was observed between Sta-Stachrom and Innovance. The results of our study suggest that Innovance, Labexpert and Sta-Stachrom are the most sensitive activity assays for detection of AT Budapest III and AT Padua I, whereas HemosIL showed considerably lower sensitivity for these two variants. As revealed in our study, the diagnostic sensitivity of AT activity assays to type II heparin binding site AT deficiency is different, and in some assays mutation dependent.

## Introduction

### Background

Antithrombin (AT) is a plasma glycoprotein that inhibits several serine proteases, including the last two of the coagulation cascade (factor Xa (FXa) and thrombin (FIIa)), and is therefore commonly known as the key endogenous anticoagulant. The inhibitory activity of AT is considerably accelerated in the presence of heparin or heparin-like substances. There are two domains on AT, which are essential for the full functioning of this serpin: (i) a reactive site involved in interaction with serine proteases, and (ii) a heparin binding site for interaction with heparin^1^.

AT deficiency is known as the most severe form of inherited thrombophilia^2^, a condition that increases the risk of developing venous thromboembolism (VTE). The complete absence of AT is incompatible with life, as demonstrated in AT knockout mice^3^. In humans, severe AT deficiency can result in embryonic lethality^4^ whereas even mildly reduced AT activity (< 80%) significantly increases the risk for VTE^5^. The prevalence of AT deficiency in the general population is estimated to be 0.02–0.17% and rises to 1–5% in patients with a history of VTE^6^.

In most cases, AT deficiency is caused by a mutation in *SERPINC1*, the gene encoding AT. AT deficiency is inherited in an autosomal dominant pattern^2^. AT deficiency was first described by Egeberg et al.^7^ and the first mutation identified in 1974 by Sas et al. was AT Budapest III^8^. Currently, there are 483 identified mutations^9^, that can cause two types of AT deficiency. Type I is manifested as decreased plasma AT concentration and, consequently, decreased plasma AT activity, whereas in type II deficiency only the activity of AT is decreased. In type II deficiency, the mutation may affect the reactive site (type IIRS), the heparin binding site (type IIHBS) or has a pleiotropic effect (type IIPE)^6^. Such categorisation is important, because the clinical presentation depends on the subtype of AT deficiency and, even within the same subtype, the phenotype may depend on the specific causative mutation. Type I usually represents a higher risk for VTE, while type II is typically associated with very heterogeneous clinical and laboratory phenotypes. Subtype IIHBS represents the most common AT deficiency in the general population. Compared with other forms of AT deficiency, type IIHBS in its heterozygous form poses a lower risk of thrombosis, while homozygous type IIHBS leads to early onset of arterial and venous thrombosis, venous thrombosis at unusual sites, and pregnancy related complications^10–13^.

Regardless of mutation type, the method of choice for detecting AT deficiency in plasma is a chromogenic assay based on inhibition of factor Xa (FXa) or thrombin (FIIa) (FXa-based and FII-based assays, respectively) in the presence of heparin. These assays are usually performed on automated coagulation analysers using commercial reagent kits. In an FIIa‐based assay that relies on endogenous AT to inhibit FIIa, FIIa and heparin are added in excess to patient plasma. The activity of uninhibited FIIa is then measured by adding a chromogenic thrombin-specific peptide substrate, which is ultimately cleaved by FIIa releasing the coloured product. The increase in absorbance detected with a spectrophotometer is inversely proportional to AT activity in the patient plasma. The FXa‐based assay is similar, except that FXa is used instead of FIIa^6^. There are many commercial AT activity assays available that differ not only in the enzyme (FXa or FIIa) but also in other reaction conditions such as enzyme source (human or bovine), incubation time, dilution, heparin concentration, buffer and pH^14^. AT deficiency is roughly defined as an AT activity level that is less than 80% of the plasma activity of the normal population^6^. Many studies have reported variable diagnostic sensitivity of AT activity assays in different AT deficiency subtypes, making laboratory diagnosis challenging^15–21^. In general, FXa-based assays are considered to be less sensitive to some IIRS mutations compared to FIIa-based assays, while some FIIa-based or FXa-based assays are better than others at detecting type IIHBS^6,22^.

AT Budapest III (p.Leu131Phe) and AT Padua I (also known as AT Rouen I, p.Arg79His), are examples of AT type IIHBS deficiency, which is reported as being challenging to detect by AT activity assays. They have a high prevalence in Europe, furthermore, AT Budapest III is also known as a founder mutation in Hungary^23^. AT Budapest III represents the typical IIHBS mutation since homozygotes develop thrombotic events at a younger age and have a higher risk of VTE than heterozygotes. AT Budapest III homozygosity is supposed to be the most severe thrombophilia (even more severe than type I AT deficiency), while cases of arterial thrombosis were also observed among heterozygotes. On the other hand, the presence of AT Padua I is in addition to VTE related also to pregnancy complications^23,24^.

To our knowledge, no meta-analysis has yet been conducted to confirm differences in the diagnostic sensitivity of FXa-based and FIIa-based assays for type IIHBS. Comprehensive data analysis from different studies could support the selection of the optimal assay, which would improve the detection of patients with AT deficiency and reduce the risk of VTE recurrence, or even prevent a first thrombotic event in asymptomatic relatives.

### Objectives

We conducted meta-analyses to investigate if any of the studied AT activity assays are better at detecting decreased AT activity in patients with AT Budapest III and AT Padua I compared with the Innovance Antithrombin Assay, one of the most commonly used AT activity assays^14^. The aim of the study was to answer the research question “Is AT activity measured with a particular AT activity assay lower than the AT activity measured with the Innovance assay in patients with AT Budapest III or AT Padua I?” Meta-analyses were performed separately for AT Budapest III and for AT Padua I.

## Results

### Study selection

**Figure 1:**
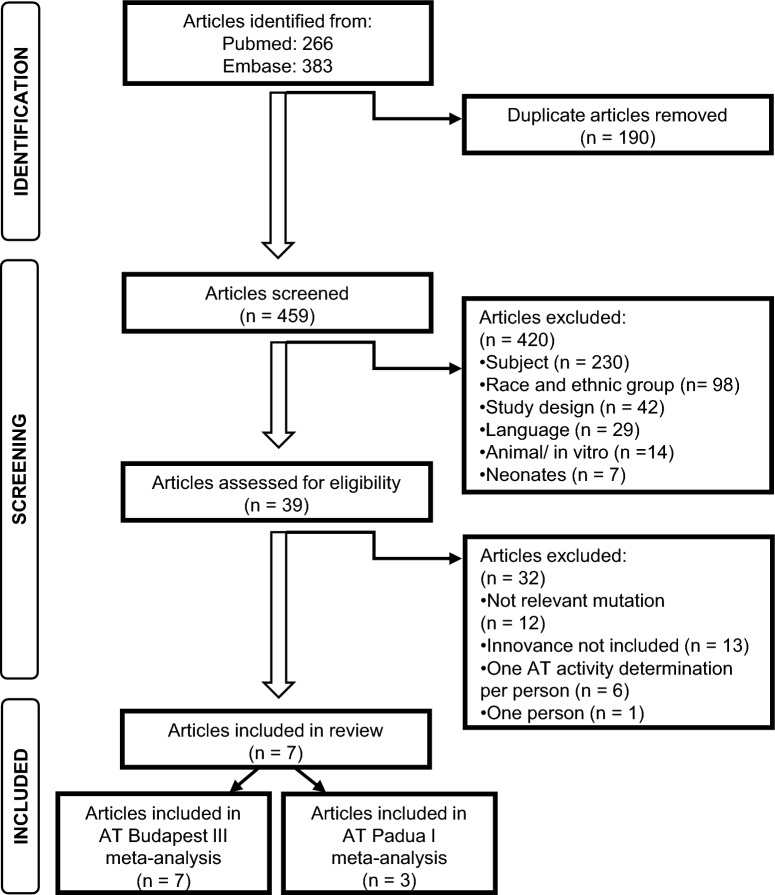
PRISMA flow diagram of included studies.

Pubmed and Embase were thoroughly searched, yielding 266 and 383 records, respectively. After removal of duplicates, 459 articles were included in the screening. 41 articles were sought for retrieval, while others were excluded because they were not in English, were performed in vitro or were animal studies, or because participants were neonates or not European and Caucasian, or because the study design was not an observative study or the study theme was not related to our topic. In eligibility assessment, 32 articles were excluded for various reasons (irrelevant mutation, Innovance assay not included, single AT activity determination per person, or only one patient included in study), while seven articles met the eligibility criteria. Seven^18,20,25–29^ and three^18,20,26^ studies were finally included in meta-analyses with respect to detection of AT Budapest III and AT Padua I, respectively (Fig. 1).

### Study characteristics

The characteristics of the studies included in the meta-analyses comparing AT activity assays with regards to measured AT activity as well as comparison of diagnostic sensitivity in patients with AT Budapest III and AT Padua I are summarised in Supplementary Tables S1 and S2, respectively. A total of four retrospective cohort studies and three cross-sectional studies were included in the meta-analyses. Patients were from Germany (2 studies), Hungary (2 studies), Serbia (2 studies) and Belgium (1 study). The number of subjects with mutation per study ranged from 2 to 89. The AT activity assays which were used in the studies besides Innovance were Berichrom (5 studies), HemosIL (2 studies), Labexpert (2 studies), Sta-Stachrom, (2 studies), Coamatic (1 study) and Biophen (1 study). The characteristics of the AT activity assays are shown in Supplementary Table S3. Three AT activity assays are FIIa-based (Berichrom, Sta-Stachrom and Biophen), whereas the remaining four are FXa-based (Innovance, HemosIL, Labexpert and Coamatic). Of these four, only Innovance contains human factor Xa, whereas the other FXa-based assays use bovine factor Xa. A single analyser was used for different AT activity assays, except in^20,28,29^ (no information on the analyser was provided). Only one study^20^ reported use of the same calibrator for all AT activity assays. Patient characteristics, such as gender, age and clinical manifestation were reported in detail by only 2 studies^25,28^.

### Assessment of study quality and risk of bias

Study quality and risk of bias for each study are represented in Supplementary Tables S4 and S5, respectively. The most frequent poorly assessed quality domains were inadequately described patient characteristics, lack of identification of confounding factors, and strategies to address them. Consequently, potential biases could be selection bias due to missing or incomplete information on patient age and gender, performance bias due to the possible presence of an acquired AT deficiency, and reporting bias due to some missing data on AT activity or inapplicable presentation of AT activity result for our meta-analysis (e.g., median AT activity with range (min–max)). None of the studies were excluded based on quality or risk of bias.

### Results of syntheses

#### Antithrombin Budapest III

##### Comparison of AT activity

Four separate meta-analyses were performed to compare Innovance with each of the four other AT activity assays (Berichrom, Sta-Stachrom, HemosIL and Labexpert) in patients with AT Budapest III.

**Figure 2:**
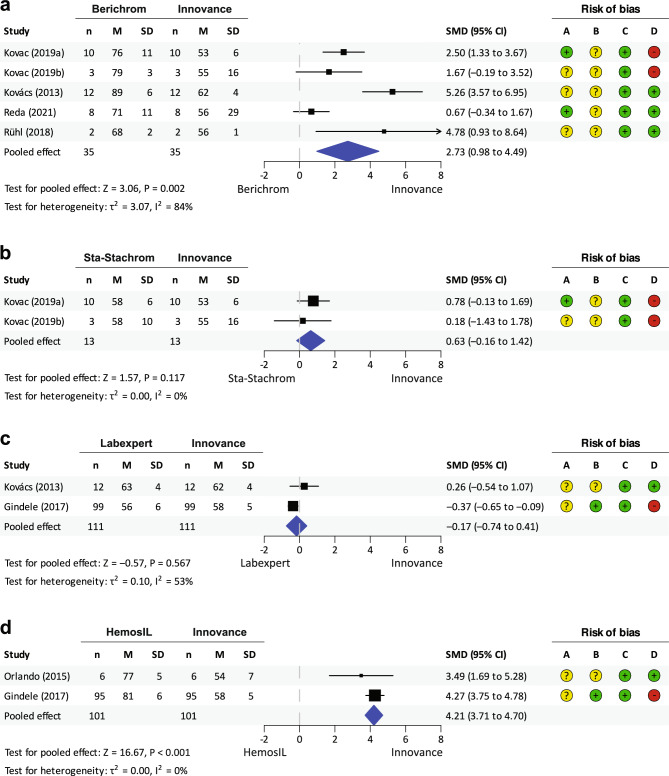
Meta-analysis comparing AT activities in patients with AT Budapest III measured with different assays and compared to Innovance: (**a**) Berichrom, (**b**) Sta-Stachrom, (**c**) Labexpert and (**d**) HemosIL. *n* number, *M* mean, *SD* standard deviation, *SMD* standardised mean difference, *CI* confidence interval; A. Selection bias; B. performance bias; C. detection bias; D. reporting bias (evaluated according to the Table S8).

The first meta-analysis that included five studies with AT activity measured by Berichrom and Innovance (Fig. 2a) indicated that AT activity measured by Berichrom is significantly higher compared to Innovance (Standardised mean difference (SMD) = 2.73, 95% CI 0.98–4.49; P = 0.002). All studies were consistent with respect to pooled effect estimate and there was also significance at the study level except for the two studies^25,29^. However, the estimated dissimilarity between studies was quite large (I^2^ = 84%).

Comparison of Innovance and Sta-Stachrom, another anti-IIa assay, revealed an insignificant, moderately higher AT activity measured with Sta-Stachrom (SMD = 0.63, 95% CI − 0.16 to 1.42; P = 0.117). Both included studies showed a consistently insignificant effect size. No heterogeneity between studies was detected (I^2^ = 0%) (Fig. 2b).

Labexpert measured lower AT activity than Innovance, however, there was also no significant difference in AT activity measured with Innovance and Labexpert at the meta-analysis level (SMD =  − 0.17, 95% CI − 0.74 to 0.41; P = 0.567). However, one of the two included studies showed a significant difference in AT activity between assays, and the included studies had opposite estimated effects. Moderate heterogeneity between studies was observed (I^2^ = 53%) (Fig. 2c).

Two studies were included in meta-analysis to compare AT activity measured by HemosIL and Innovance (Fig. 2d). AT activity measured with HemosIL was much higher compared to Innovance (SMD = 4.21, 95% CI 3.71–4.70; P < 0.001). The significant effects of the two individual studies were consistent with the pooled effect and were similar in size, with narrow confidence intervals. No substantial heterogeneity was found between studies (I^2^ = 0%).

##### Comparison of diagnostic sensitivity

Based on the data obtained, we were able to compare diagnostic sensitivity between Innovance and four other AT activity assays using Cohan’s h statistics. According to two studies, the diagnostic sensitivity of Berichrom (67% and 8%) was lower compared to Innovance (100% and 100%) in patients with AT Budapest III. The sensitivity difference was estimated as large by Cohan’s h statistics (*h* =  − 1.2^29^; *h* =  − 2.6^20^). However, two other studies did not find any sensitivity difference between these two assays (Berichrom 75%, Innovance 75%, *h* = 0.0^25^; Berichrom 100%, Innovance 100%, *h* = 0.0^27^). On the other hand, in both studies comparing Sta-Stachrom to Innovance no difference in diagnostic sensitivity was found (Sta-Stachrom 100%, Innovance 100%, *h* = 0.0^28^; Sta-Stachrom 100%, Innovance 100%, *h* = 0.0^29^) as well as in two studies comparing Labexpert to Innovance (Labexpert 100%, Innovance 100%, *h* = 0.0^20^; Labexpert 75%, Innovance 100% *h* = 0.0^26^). HemosIL appeared less sensitive compared to Innovance and the sensitivity difference is estimated as large according to two studies (HemosIL 67%, Innovance 100%, *h* =  − 1.1^18^; HemosIL 42%, Innovance 100%, *h* =  − 1.7^26^).

#### Antithrombin Padua I

##### Comparison of antithrombin activity

Two meta-analyses were performed to compare AT activity between Innovance and other AT activity assays in patients with AT Padua I.

**Figure 3:**
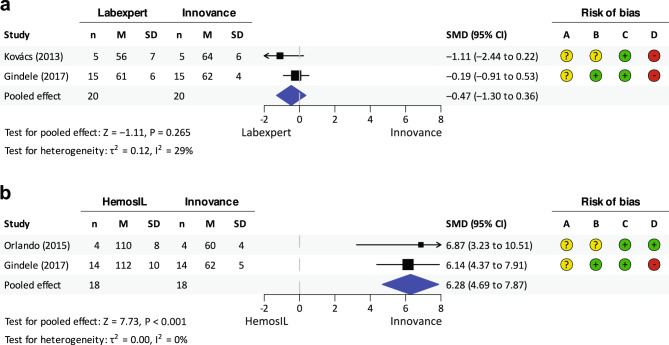
Meta-analysis comparing AT activities in patients with AT Padua I measured with different assays and compared to Innovance: (**a**) Labexpert, (**b**) HemosIL. *n* number, *M* mean, *SD* standard deviation, *SMD* standardised mean difference, *CI* confidence interval; A. Selection bias; B. performance bias; C. detection bias; D. reporting bias (evaluated according to the Table S8).

In the first one (Fig. 3a), no significant difference in AT activity between Labexpert and Innovance was detected (SMD =  − 0.47, 95% CI − 1.30 to 0.36; P = 0.265). Both included studies showed insignificantly lower AT activity measured by Labexpert compared to Innovance. A negligible heterogeneity between studies was identified (I^2^ = 29%).

The second meta-analysis (Fig. 3b) revealed significantly higher AT activity measured with HemosIL compared to Innovance (SMD = 6.28, 95% CI 4.49–7.87; P < 0.001). Both included studies had significant and very similar estimated effect sizes. No study heterogeneity was present (I^2^ = 0%).

##### Comparison of diagnostic sensitivity

Two studies that compared Labexpert and Innovance did not find any difference in diagnostic sensitivity (Labexpert 100%, Innovance 100%, *h* = 0.0^20^; Labexpert 100%, Innovance 100%, *h* = 0.0^26^). On the contrary, there was a large difference in diagnostic sensitivity of HemosIL compared to Innovance (HemosIL 0%, Innovance 100%, *h* =  − 3.1^18^; HemosIL 0%, Innovance 100%, *h* =  − 3.1^26^).

#### Overview of measured at activity and diagnostic sensitivity of all studied assays

**Figure 4:**
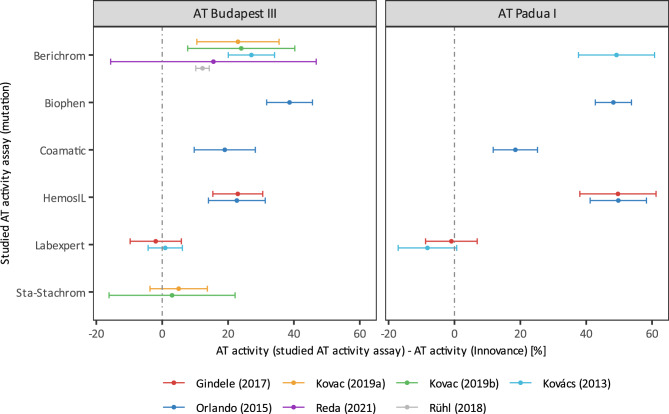
Mean differences (± SD) in AT activity between individual AT activity assays and Innovance in patients with AT Budapest III and AT Padua I as determined in different studies.

**Figure 5:**
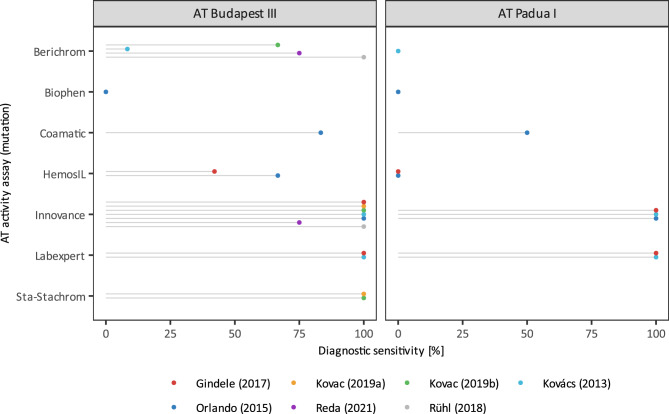
Diagnostic sensitivity of individual AT activity assays to either AT Budapest III and AT Padua I as determined in different studies meta-analysis comparing diagnostic sensitivity between Innovance and Labexpert (**a**) and HemosIL (**b**) in patients with AT Padua I.

Comparison of AT activity and diagnostic sensitivity of all seven studied AT activity assays, including those which could not be included in meta-analysis due to the insufficient number of studies that measured AT activity with a particular AT activity assay, are represented by diagrams in Figs. 4 and 5, respectively.

As shown in Fig. 4, individual mean differences in AT activity between the studied AT activity assays and Innovance are in line with the findings from meta-analyses and provide additional information about the differences in AT activity between Innovance and two other assays, namely Biophen and Coamatic. The largest differences in AT activity between the studied assays and Innovance for both mutations were observed for Berichrom, HemosIL, Biophen and Coamatic. On the other hand, the smallest difference was observed for Labexpert regardless of mutation and for Sta-Stachrom, which was not tested in patients with AT Padua I. It is also noticeable that there are differences in AT activity between mutations for particular AT activity assays. Berichrom and HemosIL apparently showed considerably different AT activity in AT Budapest III compared to AT Padua I, while the most similar AT activities in both mutations were measured with Coamatic and Labexpert. The average mean difference in measured AT activity between the studied method and Innovance exceeded 20% in three assays (Berichrom, Biophen and HemosIL) for both mutations. Since a 20% decline of normal AT activity is usually considered as AT deficiency, it is highly likely that individuals with AT activity just below 80% as measured by Innovance, would not be detected as AT deficient by methods such as Berichrom, Biophen and HemosIL.

A 20% difference in AT activity does not necessarily mean that another assay is not sensitive enough, therefore diagnostic sensitivities of different AT activity assays to both mutations were calculated for individual studies and plotted in Fig. 5. Innovance (97% and 100% sensitivity to AT Budapest III and AT Padua I, respectively), Labexpert (100% sensitivity to both mutations) and Sta-Stachrom (100% sensitivity to AT Budapest III) showed excellent sensitivity to the studied mutations*.* Sensitivity to AT Padua I was in general poorer compared to Budapest III. The largest discrepancy in sensitivity between mutations is noticed in the case of Berichrom and HemosIL. Coamatic sensitivity to AT Padua I was only 50%, while Berichrom, HemosIL and Biophen were completely unable to detect it (sensitivity 0%). The inability to detect AT Budapest III applies only to Biophen, while the Berichrom, HemosIL and Coamatic sensitivity to AT Budapest III were 61%, 54% and 83%, respectively.

### Certainty of evidence

The GRADE approach was used to assess the quality of evidence. Because of the inclusion of cross-sectional and cohort studies with direct comparison of assay results (no concern about randomisation), the certainty of the evidence of all meta-analyses was a priori assigned as high. The certainty of evidence of the meta-analysis comparing AT activity between Innovance and HemosIL in patients with AT Budapest III at the end of evaluation remained estimated as high. On the other hand, a low certainty of evidence was assessed for the meta-analysis comparing AT activity between Innovance and Labexpert in patients with AT Padua I. The certainty of evidence of four other meta-analyses was estimated as moderate. Reasons for downgrading the certainty of evidence were inconsistency between studies (2 meta-analyses) and imprecision (3 meta-analyses). Summary of findings tables are presented in Supplementary Figs. S1–S6.

There were concerns about some domains that decrease the certainty, but for which eventually the quality of the evidence was not downgraded. A risk of bias in the included studies could be present since in most studies there was incomplete information or no information about patient characteristics (gender and age) and exclusion of acquired AT deficiency. However, since we were interested in the difference in AT activity measured by two AT activity assays, the aforementioned shortcomings did not affect the results. Two studies^28,29^ also reported their outcome as median or mean with corresponding range. However, results from both compared AT activity assays were reported in the same manner and have been converted to the desired outcome (mean and standard deviation). Furthermore, some data on AT activity were missing in two studies. Since data were missing completely at random and to avoid considerable reduction in the number of subjects in case of listwise deletion, missing values were imputed with the mean of AT activities measured by particular AT activity assay. The outcome did not differ considerably in case of listwise deletion, pairwise deletion or imputation technique. Since this was not always the case for diagnostic sensitivity synthesis, listwise deletion was the most optimal choice for data included in meta-analyses comparing diagnostic sensitivity (Supplementary Table S6). Based on these assumptions, a low risk of biases was evaluated in all meta-analyses and downgrading the quality of evidence was not necessary.

There were too few studies included in each meta-analysis to assess potential publication bias with a funnel plot. However, we performed a comprehensive search strategy and there were no important concerns regarding for-profit interests in any of the included studies. Although all meta-analyses included relatively small studies, we did not downgrade for publication bias, since the number of all participants in the studies was not so small, if we consider that AT deficiency is a rare disease.

## Discussion

### Diagnostic sensitivity of AT activity assays

Based on our synthesis of data from different studies, Labexpert may represent a good substitute for Innovance as both assays give similar results (activity and diagnostic sensitivity). In fact, AT activities measured by Labexpert tend to be even lower than those measured by Innovance, although the differences are not significant. The difference in AT activity measured by Innovance and Sta-Stachrom in patients with AT Budapest III was also insignificant, although AT activity was slightly lower when measured with Innovance. The applicability of Sta-Stachrom for the detection of patients with AT Budapest III was confirmed by a similar diagnostic sensitivity in comparison to Innovance. The results of comparing diagnostic performance between Berichrom and Innovance in patients with AT Budapest III are ambiguous. AT activity measured by Berichrom is significantly higher compared to Innovance, although the effect estimate is inconclusive due to the high inconsistency between the included studies. Two studies found a large difference in diagnostic sensitivity in favour of Innovance, however, the other two studies estimated that there was no difference between these two assays. Unfortunately, a comparison of AT activity and diagnostic sensitivity between Berichrom and Innovance, as well as Sta-Stachrom and Innovance in cases with AT Padua I could not be performed, due to an insufficient number of studies. In addition, it appears that HemosIL may not be an assay of choice for detecting either AT Budapest III or AT Padua I, since AT activities measured by HemosIL are significantly higher compared to AT activities measured by Innovance. Besides this, HemosIL sensitivity to both mutations was much lower compared to Innovance. Poor sensitivity of HemosIL to these mutations and the importance of these issue is reflected by high percentage of undiagnosed patients with thrombotic event (50% AT Budapest III and 100% Padua I carriers), the highest among all AT activity assays (Table S1). Due to mainly moderate certainty of the evidence of meta-analyses, we are fairly confident in our results of comparison of AT activity. Coamatic and Biophen could not be included in meta-analyses, therefore only assumptions can be derived for these two methods. Comparison between Innovance and Coamatic as well as Innovance and Biophen revealed a relatively large difference in AT activity in both mutations. Furthermore, the diagnostic sensitivity of Coamatic for both mutations was lower compared to Innovance, while Biophen failed to detect both mutations. Therefore, normal AT activity measured by Coamatic and Biophen does not exclude the presence of AT Budapest III and AT Padua I.

The results of observational studies^18,20,26,30^ comparing AT activity assays with regards to the detection of AT Basel mutation (another common type IIHBS mutation in Europe) as well as type 1 and type 2 AT deficiency^19^ support our findings about the superiority of Innovance, Labexpet and Sta-Stachrom in the detection of type IIHBS mutations and poor sensitivity of HemosIL to this type of AT deficiency. The sensitivity of Berichrom estimated in these studies was lower than demonstrated by our results, which could be due to different mutations included in comparison.

### Effect of assay characteristics on assay diagnostic sensitivity

To the best of our knowledge, this is the first meta-analysis comparing the diagnostic performance of different AT activity assays for the detection of AT Budapest III and AT Padua I mutations in patients. Since heparin binding site is crucial for FXa inhibition, and the reactive site of the AT molecule plays a more important role in case of FIIa inhibition^31^, one would expect FXa-based assays to be more sensitive to IIHBS mutations and FIIa-based assays to IIRS mutations. The results of our meta-analyses do not support this assumption. Nevertheless, we examined diagnostic sensitivity and AT activity measured by FIIa-based assays only in one IIHBS mutation, AT Budapest III. Besides that, only two meta-analyses for FII-based assays were performed but results of one should be taken with caution due to the high inconsistency between studies. Therefore, our results regarding the efficiency of FIIa-based assays in detecting IIHBS mutations are inconclusive. On the other hand, results showing the efficiency of FXa-based assays to detect IIHBS variants are more reliable. In general, sensitivity to IIHBS mutations varied between FXa-based assays. At the same time, the sensitivity of some FXa-based assays to different IIHBS mutations also differed. Since the chosen enzyme does not show an influence to the detection of AT deficiency, other reaction factors most probably impact the assay sensitivity to a different extent depending on the specific mutation. As observed in the case of AT Budapest III and AT Padua I, the source of enzyme probably has no critical impact on assay sensitivity to a specific IIHBS mutation, since Innovance and Labexpert use human and bovine factor Xa, respectively, but nevertheless displayed similar AT activities. Moreover, Labexpert and HemosIL, both using bovine FXa, gave opposite results.

On the other hand, heparin concentration may greatly influence assay sensitivity in cases with IIHBS variants. In a study^32^ investigating differences in heparin binding affinity among different variants of IIHBS AT (AT Budapest III, AT Padua I and AT Basel) it was found that the AT Padua I protein had the lowest binding affinity to heparin and showed the slowest complex formation. Although the AT Budapest III protein was the least impaired, a weaker interaction with heparin and a slower association rate compared to the wild-type protein were observed. Furthermore, altered allosteric activation of the AT Budapest III protein was identified, however, the most severe consequences affecting the allosteric mechanism of activation and significant destabilising effects are the characteristics of the AT Padua I protein. Therefore, AT activity assays with a higher amount of heparin, favouring complex formation also in the case of diminished AT affinity towards heparin, are expected to result in a lower diagnostic sensitivity to IIHBS mutations, particularly to AT Padua I. This is also evident from our results since Innovance and Labexpert have performed better in terms of detecting decreased AT activity compared to HemosIL and Coamatic, which have at least two times higher heparin concentration.

Molecular size and source of heparin also play an important, although complex role in the sensitivity of an assay. High-affinity AT-binding pentasaccharide heparin sequence is crucial for enhancement of AT inhibitory activity, particularly for inhibition of FXa while additional 12 monosaccharides at non-reducing side of the pentasaccharide sequence are necessary for full anti-FIIa activity. Therefore, unfractionated heparin (UFH) composed of longer chains is able to inhibit FXa as well as thrombin and anti-FIIa activity increases with heparin chain length. On the other hand, LMWHs have a higher anti-FXa/anti-FIIa activity ratio and also among various low molecular weight heparins (LMWHs) significant differences in anti-FXa activity can be observed. However, because FXa binds heparin ten times weaker than FIIa, the probability that FXa dissociates from the heparin during sliding along the saccharide chain before it has reached the AT is much higher. Due to such sliding mechanism, heparins of increasing length are decreasingly active towards FXa. Also, with increasing chain length heparin inhibitory activity can be slightly reduced due to increasing interaction with plasma proteins other than AT. Furthermore, different ways of depolymerization during production can also cause different structural features of LMWHs and consequently affect their AT-mediated anticoagulant activity^33,34^. Heparins isolated from porcine intestinal mucosa tend to have higher plasma anticoagulant activity compared to heparins from bovine lung^35^. Overall, the source of heparin has probably a more pronounced effect on the sensitivity of FIIa-based assays since heparin binds directly to thrombin but not to FXa.

With regards to AT-heparin complex formation kinetics, it was already reported that shortening the incubation time regardless of the enzyme (FXa or thrombin) might improve the assay sensitivity to both type IIHBS and IIRS mutations^6^, however, this might depend on the particular mutation^18,36^. In our study incubation time itself did not determine the sensitivity of the assay. Among FIIa-based assays, Sta-Stachrom with shorter incubation time seems to be more sensitive to type IIHBS compared to Berichrom with three times longer incubation time. On the other hand, Biophen has almost the same incubation time as Sta-Stachrom, but performed much worse in detecting both mutations. In the case of FXa-based assays, the incubation time between Labexpert and Innovance differs by a factor of three, with the latter having the longest incubation time among FXa-based assays but both assays generated the lowest AT activity levels in the presence of mutation.

Last but not least, the diagnostic sensitivity of different assays can vary depending on the reaction buffer. When substitution from a basic to less basic or neutral amino acid in the heparin binding site of AT occurs as a consequence of mutation, this leads to differences in heparin binding affinity depending on the pH of the environment since complex formation is driven mainly by electrostatic interactions between negatively charged heparin and the positively charged heparin binding site on AT. AT Padua I is a missense mutation, in which more basic arginine is replaced by weakly basic histidine and consequently, the binding strength of the AT Padua I variant to heparin decreases more rapidly with higher pH compared to the wild-type AT-heparin binding. In this respect, a higher pH (> 7.4) is preferable for AT Padua I detection as the difference between the measured AT activity of mutated and wild-type AT is greater at higher pH^37^. On the other hand, pH is expected to affect assay sensitivity to AT Budapest III to a lesser extent, since the amino acid substitution (Leu to Phe) in the case of AT Budapest III does not alter the net charge of the protein. However, the pH effect cannot be supported by our results since we compared the sensitivity of different assays with only slight differences in pH. Mild quantitative AT deficiency besides the heparin binding defect observed in AT Budapest III^32^ could also contribute to easier identification of AT Budapest III in general with only minor impact of reaction conditions, which was also observed in our study.

By considering discussed assay characteristics, the reasons for lower performance of HemosIL compared to other AT activity assays included in meta-analysis may be: higher heparin concentration, type of heparin with higher affinity to type IIHBS mutations, lower enzyme activity which could lead to less coloured products and therefore falsely elevated AT activity and buffer with pH below optimal value for detection of AT Padua I.

Taken altogether, the diagnostic sensitivity to specific AT mutations or AT deficiency subtypes cannot be predicted based on a single characteristic of the AT activity assay. Therefore, appropriate combination of all reaction condition factors should be used to achieve optimal assay sensitivity.

### Strengths and potential limitations of the study

The strengths of our work are the extensive literature search and contact with authors of the included studies who provided additional information about their data.

However, our study approach does have a few limitations. Studies reported before implementation of the Innovance assay in 2009 were not included since they could not be subjected to meta-analyses. The grey literature has not been reviewed, which could contribute to the publication bias. Due to limiting the searches to publications in English language, the language bias could not be excluded. In our study, most meta-analyses included two studies and sometimes involved less than 30 patients. A small meta-analysis with a small sample size can lead to an imprecise and biased effect estimate. To emphasise, the main outcomes analysed in our study were AT activities and the aim of our meta-analyses was to compare AT activity between Innovance and other AT activity assays. Since diagnostic sensitivity is an even better indicator of the appropriateness of a particular assay for the detection of a specific mutation, we compared diagnostic sensitivities with Cohan’s h statistics. Unfortunately, due to incomplete or lacking data we were not able to compare the diagnostic sensitivity of all assays and for those that we did the sample size was much smaller.

The potential limitations of our meta-analyses that could affect the estimated effects are discussed below. The first such concern are potential differences across studies in the patient population, like age and sex, for which it is known that influence the AT activity^38,39^. In most of the examined studies, detailed information about population age and sex was not available. Since some studies did not report the exclusion of acquired AT deficiency, potentially falsely lower determined AT activities can also affect the outcome of meta-analysis. After all, there may be some inter-individual variability among patients such as different ratios of α-AT to β-AT in blood influencing heparin binding affinity^40^ and potential gene–gene or gene–environment interactions influencing AT activity. Additional factors that could contribute to the variability of results across studies are inter-laboratory differences such as type of coagulation analyser, different calibrators, reagent lots, active site concentration of coagulation proteases in reagents, assay cut-off values and staff performing the analyses. Furthermore, some missing data on AT activity were imputed with the mean of AT activities measured by particular AT activity assay. Although the imputation technique works for data missing completely at random, it cannot replace the true values. Last but not least, the way the results are presented varies between studies and therefore, can represent a possible source of variability in estimated effect between studies.

In summary, our meta-analyses have shown that Innovance and Labexpert are the most sensitive assays for the detection of both mutations, AT Budapest III and AT Padua I. Therefore, in a population where these two mutations are predominant, it would be reasonable to introduce one of these two assays into laboratory practice. In addition, good performance of Sta-Stachrom was shown for detection of AT Budapest III. Since our certainty of evidence is in general of moderate quality according to GRADE, another large cross-sectional study with the clearly defined objective of comparing different AT activity assays is highly-recommended to confirm our results.

## Methods

### Eligibility criteria

#### Types of participants

Caucasian patients of European origin, with the AT Budapest III or AT Padua I mutation confirmed by genotyping, that are symptomatic or asymptomatic, both genders, older than 1 year at the time of AT activity determination, and in which the acquired AT deficiency was excluded.

#### Types of interventions

Commercially available chromogenic AT activity assays.

#### Types of comparison

Comparison of different AT activity assays to the Innovance AT activity assay.

#### Types of outcome measures

AT activity reported as percentage of the healthy population AT activity.

#### Types of studies

Observational studies, comparing AT activity measured by different AT activity assays as the main outcome and studies, in which AT activity was determined in study participants as a secondary outcome. Only English full-text articles, published after 2009 were searched for.

Based on the described eligibility criteria, inclusion and exclusion criteria (listed in Supplementary Table S7) were created.

### Information sources and search strategy

Studies were identified by searching two electronic databases, Pubmed and Embase, and retrieving results published up to July 10, 2021. One search strategy for both mutations was conducted and later in the process of study selection, studies were divided into two mutation groups. The search strategy for each database may be found in Supplementary Information (see Supplementary Data: Search strategy). Publication type filter (Article) was used in Embase and Publication date filter (2009–present) was used in both databases to remove articles published before 2009. That year, Innovance AT assay was launched for the first time in Europe^41^.

### Selection process

The eligibility assessment for articles was performed in four main steps according to the PRISMA diagram^42^. In the first step, articles from both databases were combined and duplicates were removed. In the second step, article titles and abstracts were screened according to the predefined inclusion and exclusion criteria specified above. Those articles that met the eligibility criteria were then full-text evaluated in the third step. The second and third steps were performed independently by two reviewers. Studies were excluded based on clearly defined rationale and any inconsistency was resolved by consensus. In the fourth step, eligible studies were included in an appropriate meta-analysis.

### Data collection process

The relevant study characteristics and bibliographic data of included studies (study title, author, year of publication, study design, country, mutation, AT activity assays, AT activity (%), number of participants, age and gender of participants and clinical presentation) were extracted and collected in a predefined Excel data extraction sheet by one reviewer. The second reviewer checked the extracted data. In two cases, incomplete and missing data were used in our study as corresponding authors of those two studies have not responded to our request for additional data until the date of submission.

### Study quality and risk of bias appraisal

The quality of included studies was assessed using the Joanna Briggs Institute (JBI) Critical Appraisal Checklist for Analytical Cross-Sectional Studies^43^. Although some studies have in principle other study designs, they were appraised with the aforementioned checklist for Cross-Sectional Studies since this way, the studies could have been evaluated in terms of addressing our research question in the best possible way. The JBI checklist includes eight questions that are used to assess the study validity (design, methods and procedure) and trustworthiness of results as well as to determine the possibility of bias in its design, conduct and analysis. Question number four (“Were objective, standard criteria used for measurement of the condition?”) was omitted since it did not apply to our research question. The four main domains of bias (selection, performance, detection and reporting bias) were evaluated and the process of evaluation is explained in Supplementary Table S8. The process of study quality and risk of bias appraisal was carried out independently by two reviewers. Disagreements were resolved by discussion.

### Effect measures

Standardised mean AT activity differences were used as the primary measure of comparison between the Innovance assay and other AT activity assays for detection of AT deficiency in patients with AT Budapest III or AT Padua I. Where applicable, the difference in diagnostic sensitivity between the Innovance assay and other AT activity assays was represented as Cohan’s h as a secondary measure.

### Synthesis methods

**Figure 6:**
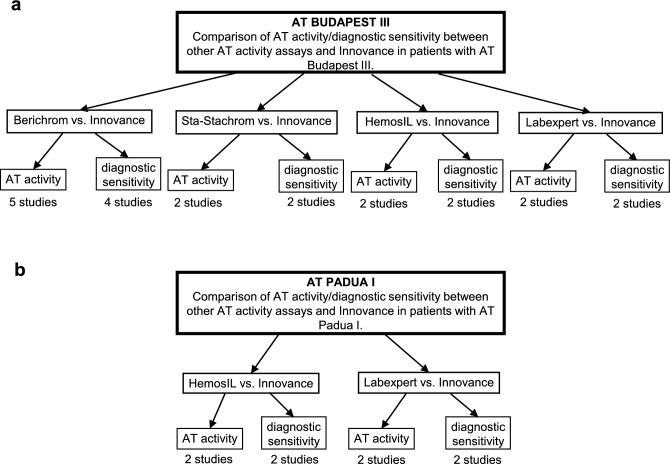
Synthesis of the results of studies with regard to mutation and AT activity assay.

The included studies were divided into groups with regards to mutation and AT activity assay, which was compared with Innovance (Fig. 6). Mean AT activity and standard deviation, as well as the diagnostic sensitivity of the assay, were calculated for each study. Where outcome was reported as median or mean and range, the mean and standard deviation were calculated using a formula developed by Wan et al.^44^. Meta-analyses using standardised mean AT activity differences were conducted for each mutation and studied AT activity assay. Meta-analyses were performed in statistical software R, using the random effect model and REML (restricted maximum likelihood estimator) approach. The results of the meta-analysis were presented with a forest plot. Statistical significance was set at ≤ 0.05. The I^2^ statistic, describing the percentage of total variation across studies that is attributable to heterogeneity rather than chance, was considered as low (I^2^ < 40%), moderate (I^2^ ≥ 40%), substantial (I^2^ ≥ 55%), or considerable (I^2^ ≥ 75%). The clinically relevant threshold was set at an absolute value of 0.2, since at this value the 85% overlap between the two distributions is large enough to make it difficult to differentiate between the groups. For each study, the difference in diagnostic sensitivity between Innovance and another AT activity assay for each mutation was represented as Cohan’s h. SMD and Cohan’s h values were considered as small (0.2–0.5) medium (0.5–0.8) or large (> 0.8) effect. In addition, individual differences in AT activity between studied assay (also the ones that could not be included in meta-analysis due to the low number of studies) and Innovance as well as diagnostic sensitivities of all AT activity assays were shown by diagram (Figs. 4, 5).

### Certainty assessment

Confidence in the body of evidence for an outcome was appraised using GRADE^45^.

### Supplementary Information

Supplementary Information 1.

Supplementary Information 2.

Supplementary Information 3.

## Data Availability

All datasets generated and/or analysed during the current study that are not included in this published article (and its Supplementary Information files) are available from the corresponding author upon reasonable request.
