# Supplementary material for: Comparison of antithrombin activity assays in detection of patients with heparin binding site antithrombin deficiency: systematic review and meta-analysis
Source: Sci Rep. 2023 Oct 4;13:16734. doi: 10.1038/s41598-023-43941-x (PMC10551003; doi:10.1038/s41598-023-43941-x)
Supplement: Supplementary file 3 — Supplementary Information 3. [file 41598_2023_43941_MOESM3_ESM.pdf]

SUPPLEMENTARY INFORMATION

Comparison of antithrombin activity assays in detection of patients with heparin binding site antithrombin deficiency: Systematic review and Meta-analysis

Tamara Rojnik[1, 2, *], Nataša Sedlar[1], Nana Turk[3], Andrej Kastrin[4], Maruša Debeljak[5,6] & Mojca Božič Mijovski[1, 2]

Affiliations

[1] Laboratory for Haemostasis and Atherothrombosis, Department of Vascular Diseases, University Medical Centre Ljubljana, 1000 Ljubljana, Slovenia.

[2] Faculty of Pharmacy, University of Ljubljana, 1000 Ljubljana, Slovenia.

[3] Central Medical Library, Faculty of Medicine, University of Ljubljana, 1000 Ljubljana, Slovenia.

[4] Institute for biostatistics and Medical Informatics, Faculty of Medicine, University of Ljubljana, 1000 Ljubljana, Slovenia.

[5] Clinical Institute for Special Laboratory Diagnostics, University Children's Hospital, University Medical Centre Ljubljana, 1000 Ljubljana, Slovenia.

[6] Department of Paediatrics, Faculty of Medicine, University of Ljubljana, 1000 Ljubljana, Slovenia.

* e-mail: tamara.rojnik@kclj.si

Supplementary Table S1: Characteristics of the studies included in the comparison of AT activity assays by meta-analyses with regards to measured AT activity and diagnostic sensitivity in patients with AT Budapest III.

ANTITHROMBIN BUDAPEST III										
**Study**	**Study design**	**Objective**	**Country**	**AT activity assay (%)**	**Coagulation analyser**	**Number of patients with mutation**	**Sex**	**Mean age (range)**	**Clinical picture**	**Number of undetected patients with thrombotic event by assay**
Reda et al., 2021 [1]	Retrospective cohort study	Assessment to what extent AT mutations impair FIIa inhibition kinetics.	Germany	Innovance (82.9 – 118.2%), Berichrom (79.4 – 112.0%)	Atellica® COAG 360 (Siemens Healthcare Diagnostics, Eschborn, Germany)	8	6F/2M	28.8 (6 – 46)	3 patients: DVT (n = 3), PE (n = 1)	Innovance (n = 1) Berichrom (n =0)
Kovać et al., 2019a [2]	Retrospective cohort study	Relationship between type of AT deficiency and pregnancy outcome.	Serbia	Innovance (83 – 119%), Berichrom (79 – 111%)	BCS XP (Siemens Healthcare Diagnostics, Marburg, Germany)	10	10F	46.9 (22 –87)	3 patients: DVT (n = 2), VTE (n = 1), pregnancy complication (n = 1)	Innovance (n = 0) Berichrom (n = cannot be determined Sta-Stachrom (n = 0)
				STA-Stachrom (80 – 120%)	STA Compact coagulation analyser					
Kovać et al., 2019b [3]	Retrospective cohort study	Evaluation of genotype phenotype correlation in AT deficiency in a paediatric population.	Serbia	Innovance (83 – 119%), Berichrom (79 – 111%)	BCS XP (Siemens Healthcare Diagnostics, Marburg, Germany)	3	3M	NA	1 patient: DVT (n =1), superficial VTE (n = 2)	Innovance (n = 0) Berichrom (n = 0) Sta-Stachrom (n = 0)
				STA-Stachrom (80 – 120%)	STA Compact coagulation analyser					

ANTITHROMBIN BUDAPEST III										
**Study**	**Study design**	**Objective**	**Country**	**AT activity assay (%)**	**Coagulation analyser**	**Number of patients with mutation**	**Sex**	**Mean age (range)**	**Clinical picture**	**Number of undetected patients with thrombotic event by assay**
Rühl et al., 2018 [4]	Cross-sectional study	The effect of anticoagulant drugs on diagnostic efficiency of AT activity assays.	Germany	Innovance (82.9 – 118.2%), Berichrom (79.4 – 112.0%)	BCS XP (Siemens Healthcare Diagnostics, Eschborn, Germany)	2	NA	NA	NA	NA
Gindele et al., 2017 [5]	Retrospective cohort study	Clinical, laboratory and genetic characteristics of patients with AT deficiency and comparison of diagnostic efficiency of AT activity assays.	Hungary	Innovance (80 – 120%)	BCS-XP (Siemens Healthcare Diagnostics, Eschborn, Germany)	89	36F/42M Data are incomplete.	31.14 (5 – 68) Data are incomplete	40 patients: DVT (n = 33), ATE (n = 7), PE (n = 3) Data are incomplete.	Innovance (n = 0/33 patients) HemosIL (n = 4/8 patients) Labexpert (n = 0/28 patients) Data are incomplete.
				HemosIL (80 – 120%)		25				
				Labexpert (80 – 120%)		85				
Orlando et al, 2015 [6]	Cross-sectional study	Comparison of diagnostic efficiency of AT activity assays in patients with type IIHBS AT deficiency.	Belgium	Innovance (80 – 120%), Biophen (80 – 120%), Coamatic (80 – 120%), HemosIL (80 – 120%)	ACL TOP500 (Instrumentation Laboratory)	6	NA	NA	NA	NA

ANTITHROMBIN BUDAPEST III										
**Study**	**Study design**	**Objective**	**Country**	**AT activity assay (%)**	**Coagulation analyser**	**Number of patients with mutation**	**Sex**	**Mean age (range)**	**Clinical picture**	**Number of undetected patients with thrombotic event by assay**
Kovács et al., 2013 [7]	Cross-sectional study	Comparison of diagnostic efficiency of anti-FIIa and anti-FXa AT assays in patients with type II HBS AT deficiency	Hungary	Innovance (> 80%), Berichrom (> 80%), Labexpert (> 80%) The same calibrator used.	NA	12	7F/5M	NA	NA	NA

Abbreviations: M, male; F, female; DVT, deep vein thrombosis; PE, pulmonary embolism; VTE, venous thromboembolism; ATE, arterial thrombosis; NA, not available; n, number.

Supplementary Table S2: Characteristics of the studies included in the comparison of AT activity assays by meta-analyses with regards to measured AT activity and diagnostic sensitivity in patients with AT Padua I.

**ANTITHROMBIN PADUA I**										
**Study**	**Study design**	**Objective**	**Country**	**AT activity assay (%)**	**Coagulation analyser**	**Number of patients with mutation**	**Sex**	**Mean age (range)**	**Clinical picture**	**Number of undetected patients with thrombotic event by assay**
Gindele et al., 2017 [5]	Retrospective cohort study	Clinical, laboratory and genetic characteristics of patients with AT deficiency and comparison of diagnostic efficiency of AT activity assays.	Hungary	Innovance (80 – 120%)	BCS-XP (Siemens Healthcare Diagnostics, Eschborn, Germany)	14	12F/3M	37.2 (16 – 66)	4 patients: DVT (n = 2), ATE (n = 1), PE (n =1)	Innovance (n = 0) HemosIL (n = 3/3 patients) Labexpert (n = 0) Data are incomplete.
				HemosIL (80 – 120%)		10				
				Labexpert (80 – 120%)		14				
Orlando et al, 2015 [6]	Cross-sectional study	Comparison of diagnostic efficiency of AT activity assays in patients with type IIHBS AT deficiency.	Belgium	Innovance (80 – 120%)	ACL TOP500 (Instrumentation Laboratory)	4	NA	NA	NA	NA
				Biophen (80 – 120%)		3				
				Coamatic (80 – 120%)		4				
				HemosIL (80 – 120%)		4				
Kovács et al., 2013 [7]	Cross-sectional study	Comparison of diagnostic efficiency of anti-FIIa and anti-FXa AT assays	Hungary	Innovance (> 80%)	NA	4	3F/1M	NA	NA	NA
				Berichrom (> 80%)		5	4F/1M			

		in patients with type II HBS AT deficiency		Labexpert (> 80%) The same calibrator used.		5	4F/1M			

Abbreviations: M, male; F, female; DVT, deep vein thrombosis; PE, pulmonary embolism; ATE, arterial thrombosis; NA, not available; n, number.

Supplementary Table S3: Detailed overview of AT activity assays used in studies included in meta-analyses.

AT activity assay	Enzyme	Origin of enzyme	Amount of enzyme	Substrate	Incubation time*	Amount of heparin	Predilution*	Final dilution (method)*	Buffer	Analyser
Innovance® Antithrombin assay, Siemens	FXa	human	1000 U/L	benzyloxycarbonyl-D-Leu-Gly-Arg-ANBA-methylamide-acetate	180-190 s	1500 U/L	1:4	1:80 (kinetic method)	Tris/HCl, pH 8.0	Siemens BCS-System
Berichrom® Antithrombin III (A) assay, Siemens	FIIa	bovine	NA	Tos-Gly-Pro-Arg-ANBA-IPA	180-300 s	NA	1:3	1:72 (kinetic method)	Tris/HCI, NaCl, pH 8.2	Siemens BCS-System
HemosIL® liquid antithrombin, Werfen	FXa	bovine	10.4 nkat/mL	N-α-Z-D-Arg-Gly-Arg-pNA	100-140 s	NA (3000 U/L) [8]	3:95	6:589 (kinetic method)	NA	IL Coagulation Systems
Labexpert antithrombin H+P, Labexpert Ltd	FXa	bovine	12 nkat/mL	Suc-Ile-Glu-(γPip)Gly-Arg-pNA	60 s	1000 U/L	1:50	1:150 (kinetic method)	Tris/HCl pH 8.4	Siemens BCS XP System and Ceveron, Technoclone
Biophen™ AT (Anti-IIa), Hyphen	FIIa	bovine	NA	H-D-Phe-Pip-Arg-pNa	50 s	NA	1:4	3:100 (kinetic method)	pH 8.40	Siemens BCS System
Coamatic® Antithrombin, Chromogenix**	FXa	bovine	2.9 nkat/mL	N-α-Cbo-d-Arg-Gly-Arg-pNA	90 s	NA (5000 U/L) [8]	1:121	1:363 (kinetic method)	Tris buffer pH 8.2	Microplate method
Sta-Stachrom ATIII, Diagnostica Stago	FIIa	bovine	11.3 nkat/mL	EtM-SPro-Arg-pNa,AcOH	60 s	NA	1:20	1:60 (kinetic method)	Sta® - Owren-Koller pH 7.35	Analyser of Sta® line

***** Assay characteristics, especially incubation time, predilution and final dilution may differ according to the analyser. Assay characteristics presented in the table are related to the analyser on which the method was originally validated or to the most commonly used analyser in studies in case of multiple validated protocols.

** Coamatic Antithrombin is no longer available in Europe since 2020.

NA, not available

Supplementary Table S4: Appraisal of quality of individual studies.

Study	Study quality							
	**Were the criteria for inclusion in the sample clearly defined?**	**Were the study subjects and the setting described in detail?**	**Was the exposure (presence of mutation) measured in a valid and reliable way?**	**Were confounding factors identified?**	**Were strategies to deal with confounding factors stated?**	**Were the outcomes (AT activity) measured in a valid and reliable way?**	**Was appropriate statistical analysis used?**	**Comment**
Reda et al., 2021 [1]	yes	yes	unclear[1]	unclear[2]	yes	yes	yes	[1]There is no information about how exposure was measured. [2]Only therapy was excluded.
Kovac et al., 2019a [2]	yes	yes	yes	no	n.a.	yes	yes	
Kovac et al., 2019b [3]	yes	unclear[1]	yes	unclear[2]	unclear [3]	yes	yes	[1]There is no information on patient age. [2]Only liver disease and nephrotic syndrome are excluded. [3]It is unclear if determination of AT activity was performed for one patient while on anticoagulant therapy.
Rühl et al., 2018 [4]	yes	unclear[1]	unclear[2]	unclear[3]	yes	yes	yes	[1]There is no information on patient age and sex.

Study	Study quality							
	**Were the criteria for inclusion in the sample clearly defined?**	**Were the study subjects and the setting described in detail?**	**Was the exposure (presence of mutation) measured in a valid and reliable way?**	**Were confounding factors identified?**	**Were strategies to deal with confounding factors stated?**	**Were the outcomes (AT activity) measured in a valid and reliable way?**	**Was appropriate statistical analysis used?**	**Comment**
								[2]There is no information about how exposure was measured. [3]Only therapy was excluded.
Gindele et al., 2017 [5]	yes	unclear[1]	yes	yes	unclear[2]	yes	yes	[1]There is no information on patient age. [2]There is no information about how these patients were treated.
Orlando et al, 2015 [6]	yes	unclear[1]	yes	no	n.a.	yes	yes	[1]There is no information on patient age and sex.
Kovács et al., 2013 [7]	yes	unclear[1]	yes	unclear[2]	yes	yes	unclear[3]	[1]There is no information on patient age. [2]Only acute thrombosis is excluded. [3]There is no information on the statistics used.

Abbreviations: n.a., Not applicable.

Supplementary Table S5: Risk of bias appraisal for individual studies

STUDY/BIAS	Selection bias	Performance bias	Detection bias	Reporting bias
Reda et al., 2021 [1]				
Kovac et al., 2019a [2]				
Kovac et al., 2019b [3]				
Rühl et al., 2018 [4]				
Gindele et al., 2017 [5]				
Orlando et al, 2015 [6]				
Kovács et al., 2013 [7]				AT Budapest III / AT Padua I

Risk of bias: low (green), medium (yellow), high (red).

Supplementary Figure S1: Summary of findings' table: Comparison of AT activity in patients with AT Budapest III between Innovance and Berichrom.

Summary of findings:

Comparison of antithrombin activity between Berichrom and Innovance in patients with AT Budapest III

Patient or population: patients with AT Budapest III
Setting: clinical setting
Intervention: Berichrom
Comparison: Innovance

Outcomes	Anticipated absolute effects* (95% CI) Risk with Innovance	Anticipated absolute effects* (95% CI) Risk with Berichrom	Relative effect (95% CI)	№ of participants (studies)	Certainty of the evidence (GRADE)	Comments
Antithrombin activity	-	SMD **2.73 SD higher** (0.98 higher to 4.49 higher)	-	70 (5 observational studies)	⊕⊕⊕◯ Moderate[a,b]	Antithrombin activity measured by Berichrom was significantly higher compared to Innovance. SMD 2.73 corresponds to a large difference in antithrombin activity between these two methods. Antithrombin activity measured by Berichrom is likely much higher compared to Innovance in patients with AT Budapest III.

***The risk in the intervention group** (and its 95% confidence interval) is based on the assumed risk in the comparison group and the **relative effect** of the intervention (and its 95% CI).

CI: confidence interval; **SMD:** standardised mean difference

GRADE Working Group grades of evidence
High certainty: we are very confident that the true effect lies close to that of the estimate of the effect.
Moderate certainty: we are moderately confident in the effect estimate: the true effect is likely to be close to the estimate of the effect, but there is a possibility that it is substantially different.
Low certainty: our confidence in the effect estimate is limited: the true effect may be substantially different from the estimate of the effect.
Very low certainty: we have very little confidence in the effect estimate: the true effect is likely to be substantially different from the estimate of effect.

Explanations

a. Inconsistency: 1) I^2= 84% indicates considerable heterogeneity of the studies; 2) large differences in size effects between studies
b. Large effect: despite the large pooled effect, we did not increase the quality due to inconsistency between studies regarding effect size

Supplementary Figure S2: Summary of findings' table: Comparison of AT activity in patients with AT Budapest III between Innovance and Sta-Stachrom.

Summary of findings:

Comparison of antithrombin activity between Sta-Stachrom and Innovance in patients with AT Budapest III

Patient or population: patients with AT Budapest III
Setting: clinical setting
Intervention: Sta-Stachrom
Comparison: Innovance

Outcomes	**Anticipated absolute effects*** (95% CI)		Relative effect (95% CI)	№ of participants (studies)	Certainty of the evidence (GRADE)	Comments
	**Risk with Innovance**	**Risk with Sta-Stachrom**				
Antithrombin activity	-	SMD **0.63 SD higher** (0.16 lower to 1.42 higher)	-	26 (2 observational studies)	⨁⨁⨁◯ Moderate[a]	Antithrombin activity measured by Sta-Stachrom was not significantly higher compared to Innovance. SMD 0.63 corresponds to a moderate difference in antithrombin activity between these two methods. There is likely no difference in antithrombin activity between Sta-Stachrom and Innovance in patients with AT Budapest III.

***The risk in the intervention group** (and its 95% confidence interval) is based on the assumed risk in the comparison group and the **relative effect** of the intervention (and its 95% CI).

CI: confidence interval; **SMD:** standardised mean difference

GRADE Working Group grades of evidence
High certainty: we are very confident that the true effect lies close to that of the estimate of the effect.
Moderate certainty: we are moderately confident in the effect estimate: the true effect is likely to be close to the estimate of the effect, but there is a possibility that it is substantially different.
Low certainty: our confidence in the effect estimate is limited: the true effect may be substantially different from the estimate of the effect.
Very low certainty: we have very little confidence in the effect estimate: the true effect is likely to be substantially different from the estimate of effect.

Explanations

a. Imprecision: CI cross a prespecified threshold (0.2), probably due to small sample size (< 30).

Supplementary Figure S3: Summary of findings' table: Comparison of AT activity in patients with AT Budapest III between Innovance and Labexpert.

Summary of findings:

Comparison of antithrombin activity between Labexpert and Innovance in patients with AT Budapest III

Patient or population: patients with AT Budapest III
Setting: clinical setting
Intervention: Labexpert
Comparison: Innovance

Outcomes	Anticipated absolute effects* (95% CI)		Relative effect (95% CI)	№ of participants (studies)	Certainty of the evidence (GRADE)	Comments
	**Risk with Innovance**	**Risk with Labexpert**				
Antithrombin activity	-	SMD **0.17 SD lower** (0.74 lower to 0.41 higher)	-	222 (2 observational studies)	⊕⊕⊕◯ Moderate[a,b]	Antithrombin activity measured by Labexpert was not significantly lower compared to Innovance. SMD 0.17 corresponds to a small difference in antithrombin activity between these two methods. There is likely no difference in antithrombin activity between Labexpert and Innovance in patients with AT Budapest III.

***The risk in the intervention group** (and its 95% confidence interval) is based on the assumed risk in the comparison group and the **relative effect** of the intervention (and its 95% CI).

CI: confidence interval; **SMD:** standardised mean difference

GRADE Working Group grades of evidence
High certainty: we are very confident that the true effect lies close to that of the estimate of the effect.
Moderate certainty: we are moderately confident in the effect estimate: the true effect is likely to be close to the estimate of the effect, but there is a possibility that it is substantially different.
Low certainty: our confidence in the effect estimate is limited: the true effect may be substantially different from the estimate of the effect.
Very low certainty: we have very little confidence in the effect estimate: the true effect is likely to be substantially different from the estimate of effect.

Explanations

a. Inconsistency: I^2 =53% (moderate heterogeneity), however quality of evidence was not downgraded for inconsistency since other indicators of heterogeneity were not present.
b. Imprecision: CI cross a prespecified threshold (0.2)

Supplementary Figure S4: Summary of findings' table: Comparison of AT activity in patients with AT Budapest III between Innovance and HemosIL.

Summary of findings:

Comparison of antithrombin activity between HemosIL and Innovance in patients with AT Budapest III

Patient or population: patients with AT Budapest III
Setting: clinical setting
Intervention: HemosIL
Comparison: Innovance

Outcomes	Anticipated absolute effects* (95% CI) Risk with Innovance	Risk with HemosIL	Relative effect (95% CI)	№ of participants (studies)	Certainty of the evidence (GRADE)	Comments
Antithrombin activity	-	SMD **4.21 SD higher** (3.71 higher to 4.7 higher)	-	202 (2 observational studies)	⊕⊕⊕⊕ High[a]	Antithrombin activity measured by HemosIL was significantly higher compared to Innovance. SMD 4.21 corresponds to a large difference in antithrombin activity between these two methods. Antithrombin activity measured by HemosIL is very likely much higher compared to Innovance in patients with AT Budapest III.

***The risk in the intervention group** (and its 95% confidence interval) is based on the assumed risk in the comparison group and the **relative effect** of the intervention (and its 95% CI).

CI: confidence interval; **SMD:** standardised mean difference

GRADE Working Group grades of evidence
High certainty: we are very confident that the true effect lies close to that of the estimate of the effect.
Moderate certainty: we are moderately confident in the effect estimate: the true effect is likely to be close to the estimate of the effect, but there is a possibility that it is substantially different.
Low certainty: our confidence in the effect estimate is limited: the true effect may be substantially different from the estimate of the effect.
Very low certainty: we have very little confidence in the effect estimate: the true effect is likely to be substantially different from the estimate of effect.

Explanations

a. Large effect: large pooled effect size; both included studies are consistent in estimate of effect size

Supplementary Figure S5: Summary of findings' table: Comparison of AT activity in patients with AT Padua I between Innovance and Labexpert.

Summary of findings:

Comparison of antithrombin activity between Labexpert and Innovance in patients with AT Padua I

Patient or population: patients with AT Padua I
Setting: clinical setting
Intervention: Labexpert
Comparison: Innovance

Outcomes	**Anticipated absolute effects*** (95% CI)		Relative effect (95% CI)	№ of participants (studies)	Certainty of the evidence (GRADE)	Comments
	**Risk with Innovance**	**Risk with Labexpert**				
Antithrombin activity	-	SMD **0.47 SD lower** (1.3 lower to 0.36 higher)	-	40 (2 observational studies)	⊕⊕◯◯ Low[a,b]	Antithrombin activity measured by Labexpert was not significantly lower compared to Innovance. SMD 0.47 corresponds to a moderate difference in antithrombin activity between these two methods. It is unlikely no difference in antithrombin activity between Labexpert and Innovance in patients with AT Padua I.

***The risk in the intervention group** (and its 95% confidence interval) is based on the assumed risk in the comparison group and the **relative effect** of the intervention (and its 95% CI).

CI: confidence interval; **SMD:** standardised mean difference

GRADE Working Group grades of evidence
High certainty: we are very confident that the true effect lies close to that of the estimate of the effect.
Moderate certainty: we are moderately confident in the effect estimate: the true effect is likely to be close to the estimate of the effect, but there is a possibility that it is substantially different.
Low certainty: our confidence in the effect estimate is limited: the true effect may be substantially different from the estimate of the effect.
Very low certainty: we have very little confidence in the effect estimate: the true effect is likely to be substantially different from the estimate of effect.

Explanations

a. Inconsistency: I^2=29% (negligeble heterogeneity), however there is large difference in estimated size effects between studies
b. Imprecision: CI cross a prespecified threshold (0.2), probably due to small sample size (< 30).

Supplementary Figure S6: Summary of findings' table: Comparison of AT activity in patients with AT Padua I between Innovance and HemosIL.

Summary of findings:

Comparison of antithrombin activity between HemosIL and Innovance in patients with AT Padua I

Patient or population: patients with AT Padua I
Setting: clinical setting
Intervention: Hemosil
Comparison: Innovance

Outcomes	Anticipated absolute effects* (95% CI)		Relative effect (95% CI)	№ of participants (studies)	Certainty of the evidence (GRADE)	Comments
	**Risk with Innovance**	**Risk with Hemosil**				
Antithrombin activity	-	SMD **6.28 SD higher** (4.69 higher to 7.87 higher)	-	36 (2 observational studies)	⊕⊕⊕◯ Moderate[a,b]	Antithrombin activity measured by HemosIL was significantly higher compared to Innovance. SMD 6.28 corresponds to a large difference in antithrombin activity between these two methods. Antithrombin activity measured by HemosIL is likely much higher compared to Innovance in patients with AT Padua I.

***The risk in the intervention group** (and its 95% confidence interval) is based on the assumed risk in the comparison group and the **relative effect** of the intervention (and its 95% CI).

CI: confidence interval; **SMD:** standardised mean difference

GRADE Working Group grades of evidence
High certainty: we are very confident that the true effect lies close to that of the estimate of the effect.
Moderate certainty: we are moderately confident in the effect estimate: the true effect is likely to be close to the estimate of the effect, but there is a possibility that it is substantially different.
Low certainty: our confidence in the effect estimate is limited: the true effect may be substantially different from the estimate of the effect.
Very low certainty: we have very little confidence in the effect estimate: the true effect is likely to be substantially different from the estimate of effect.

Explanations

a. Imprecision: small sample size (< 30)
b. Large effect: despite the large pooled effect, we did not increase the quality due to small sample size

Supplementary Table S6: Comparison of AT activity and diagnostic sensitivity of AT activity assays to Innovance after listwise deletion, pairwise deletion and imputation technique for the data that were missing (incomplete) in different studies.

Mutation		AT Budapest III			AT Padua I			AT Budapest III			AT Padua I		
Study	Compared AT activity assays /Missing data analysis	Listwise (AT activity (%))	Pairwise (AT activity (%))	Imputation (AT activity (%))	Listwise (AT activity (%))	Pairwise (AT activity (%))	Imputation (AT activity (%))	Listwise (diagnostic sensitivity (%))	Pairwise (diagnostic sensitivity (%))	Imputation (diagnostic sensitivity (%))	Listwise (diagnostic sensitivity (%))	Pairwise (diagnostic sensitivity (%))	Imputation (diagnostic sensitivity (%))
Gindele et. al., 2017 [5]	Innovance	57 (n = 19)	58 (n = 89)	58 (n = 95)	62 (n = 10)	62 (n = 14)	62 (n = 14)	100 (n = 19)	100 (n = 89)	100 (n = 95)	100 (n = 10)	100 (n = 14)	100 (n = 14)
	HemosIL	81 (n = 19)	81 (n = 25)	81 (n = 95)	112 (n = 10)	112 (n = 10)	112 (n = 14)	42 (n = 19)	44 (n = 25)	12 (n = 95)	0 (n = 10)	0 (n = 10)	0 (n = 14)
Gindele et al., 2017 [5]	Innovance	58 (n = 75)	58 (n = 89)	58 (n = 99)	63 (n = 13)	62 (n = 14)	62 (n = 15)	100 (n = 75)	100 (n = 89)	100 (n = 99)	100 (n = 13)	100 (n = 14)	100 (n = 15)
	Labexpert	56 (n = 75)	56 (n = 85)	56 (n = 99)	62 (n = 13)	61 (n = 14)	61 (n = 15)	100 (n = 75)	100 (n = 85)	100 (n = 99)	100 (n = 13)	100 (n = 14)	100 (n = 15)
Kovacs et al., 2013 [7]	Innovance				64 (n = 4)	64 (n = 4)	64 (n = 5)				100 (n = 4)	100 (n = 4)	100 (n = 5)
	Labexpert				57 (n = 4)	56 (n = 5)	56 (n = 5)				100 (n = 4)	100 (n = 5)	100 (n = 5)

Supplementary Table S7: Inclusion and exclusion criteria.

	Inclusion criteria	Exclusion criteria
P	Caucasians from Europe	< 1 year old at the time of AT activity determination
	Presence of AT Budapest III or AT Padua I (Rouen I) mutations, confirmed by genotyping	Acquired AT deficiency (liver dysfunction, recent surgery, acute thrombosis, DIC, cancer, L-asparaginase therapy, preeclampsia, nephrotic syndrome, oestrogen therapy, pregnancy, postpartum, active inflammatory bowel disease, anticoagulant therapy (except vitamin K antagonists), haemodialysis, plasmapheresis, cardiopulmonary bypass)
I	AT activity measured by at least one of the commercial AT activity assays	Lack of reference range for particular AT activity assay<br>Only β-antithrombin activity given
C	AT activity measured by Innovance activity assay	
O	Observational studies	Conference abstracts, letters, expert opinions, correspondences
	Full-text English articles	Articles published before 2009

Supplementary Data: Search strategy.

PUBMED (747)

(("Antithrombin III Deficiency" [mesh] OR "Antithrombin III" [mesh] OR "Antithrombin deficiency" [tiab] OR "SERPINC1" [tiab] OR "ATBp3" [tiab] OR "antithrombin Budapest" [tiab] OR "antithrombin III Budapest" [tiab] OR "Leu131Phe" [tiab] OR "antithrombin Padua" [tiab] OR "antithrombin III Padua" [tiab] OR "Arg79His" [tiab]) AND (("antithrombin assay*" [tiab] OR "antithrombin activit*" [tiab] OR "activity assay*" [tiab] OR "anti-FIIa" [tiab] OR "anti-Fxa" [tiab] OR "factor Xa-based" [tiab] OR "thrombin-based" [tiab]) OR ("Mutation" [Mesh] OR "Mutation*" [tiab] OR "Genetics" [Mesh]))) NOT ("review" [Publication Type] OR "review literature as topic" [MeSH Terms] OR "systematic review" [Publication Type] OR "systematic review" [tiab] OR "review" [tiab] OR "meta-analysis" [Publication Type] OR "meta-analysis as topic" [MeSH Terms] OR "Network Meta-Analysis" [MeSH] OR "meta-analysis" [tiab] OR "editorial" [Publication Type] OR "editorial" [tiab] OR "comment" [Publication Type] OR "letter" [Publication Type]).

Records: 747

Filters: Publication date filter (2009-present)

Articles after Publication date filter was used: 266

EMBASE (1791)

1 exp antithrombin deficiency/

2 antithrombin deficiency.ti,ab.

3 exp antithrombin III/

4 SERPINC1.ti,ab.

5 ATBp3.ti,ab.

6 antithrombin Budapest.ti,ab.

7 antithrombin III Budapest.ti,ab.

8Leu131Phe.ti,ab.

9 antithrombin Padua.ti,ab.

10 antithrombin III Padua.ti,ab.

11 antithrombin assay$.ti,ab.

12 antithrombin activit$.ti,ab.

13 activity assay$.ti,ab.

14 anti-FIIa.ti,ab.

15 anti-FXa.ti,ab.

16 factor Xa-based.ti,ab.

17 thrombin-based.ti,ab.

18 mutation$.ti,ab.

19 genetics.ti,ab.

20 1 OR 2

21 3 OR 4 OR 5 OR 6 OR 7 OR 8 OR 9 OR 10 OR 11

22 20 OR 21

23 11 OR 12 OR 13 OR 14 OR 15 OR 16 OR 17

24 18 OR 19

25 23 OR 24

26 22 AND 25

Records: 1791

Filters: Publication date (2009-present) and publication type filter (Article)

Articles after Publication type filter was used:897

Articles after Publication date filter was used: 383

Supplementary Table S8: Risk of bias evaluation.

Type of bias	Criteria for bias exclusion	Are these criteria met?				
		Low risk of bias			High risk of bias	
		Yes	Probably yes	No information	Probably no	No
Selection bias	A clear description of population characteristics (country, period, age, sex, clinical picture).<br>Objectively confirmed mutation. Consecutively selected patients.					
Performance bias	Identification of confounding factors (acquired AT deficiency) and detailed description of proceedings.					
Detection bias	Outcomes measured validly and reliably.					
Reporting bias	AT activity reported in percentages for each individual or as mean and standard deviation for a group of patients.					

REFERENCES

1 Reda, S. *et al.* Functional Characterization of Antithrombin Mutations by Monitoring of Thrombin Inhibition Kinetics. *Int J Mol Sci*. **22**, (2021).

2 Kovac, M. *et al.* The influence of specific mutations in the AT gene (SERPINC1) on the type of pregnancy related complications. *Thromb Res*. **173**, 12-19 (2019).

3 Kovac, M. *et al.* Genotype phenotype correlation in a pediatric population with antithrombin deficiency. *Eur J Pediatr*. **178**, 1471-1478 (2019).

4 Ruhl, H., Reda, S., Muller, J., Oldenburg, J. & Potzsch, B. Activated Factor X-Based versus Thrombin-Based Antithrombin Testing in Thrombophilia Workup in the DOAC Era. *Thromb Haemost*. **118**, 381-387 (2018).

5 Gindele, R. *et al.* Clinical and laboratory characteristics of antithrombin deficiencies: A large cohort study from a single diagnostic center. *Thromb Res*. **160**, 119-128 (2017).

6 Orlando, C., Heylen, O., Lissens, W. & Jochmans, K. Antithrombin heparin binding site deficiency: A challenging diagnosis of a not so benign thrombophilia. *Thromb Res*. **135**, 1179-1185 (2015).

7 Kovacs, B. *et al.* The superiority of anti-FXa assay over anti-FIIa assay in detecting heparin-binding site antithrombin deficiency. *Am J Clin Pathol*. **140**, 675-679 (2013).

8 Bereczky, Z., Gindele, R., Speker, M. & Kallai, J. Deficiencies of the Natural Anticoagulants - Novel Clinical Laboratory Aspects of Thrombophilia Testing. *EJIFCC*. **27**, 130-146 (2016).
